# Pheochromocytoma presenting with achalasia: exploring a possible link

**DOI:** 10.1210/jcemcr/luag168

**Published:** 2026-07-16

**Authors:** Gitanjali Reddy, Julius Wu, Zhanna Zavgorodneva, Samy I McFarlane

**Affiliations:** SUNY Downstate Health Sciences University, College of Medicine, Brooklyn, NY 11203, USA; SUNY Downstate Health Sciences University, College of Medicine, Brooklyn, NY 11203, USA; Department of Endocrinology, Diabetes, and Metabolism, SUNY Downstate Health Sciences University, Brooklyn, NY 11203, USA; Department of Endocrinology, Diabetes, and Metabolism, SUNY Downstate Health Sciences University, Brooklyn, NY 11203, USA

**Keywords:** pheochromocytoma, achalasia, catecholamine excess, esophageal dysmotility, adrenal incidentaloma

## Abstract

Although the adrenergic triad is traditionally regarded as the classic manifestation of pheochromocytoma, an increasing proportion of cases are now incidentally identified during evaluation for co-occurring conditions. To our knowledge, no published reports have described the copresentation of achalasia and a pheochromocytoma. A 62-year-old woman presented with several months of progressive cough, intermittent nonbloody, nonbilious emesis, dysphagia, and unintentional weight loss. Computed tomography of the chest, abdomen, and pelvis demonstrated multifocal pneumonia, achalasia, and an incidental 5.7-cm left adrenal mass. High-resolution esophageal manometry confirmed type II achalasia. The diagnosis of pheochromocytoma was established based on characteristic imaging features and markedly elevated plasma normetanephrine levels—approximately 30-fold above the upper limit of normal. Following adrenalectomy, biochemical values normalized, and her achalasia-related symptoms significantly improved. Although it could be merely a co-occurrence of achalasia and pheochromocytoma, the improvement in achalasia symptoms after adrenalectomy suggests that elevated catecholamines may contribute to or exacerbate esophageal motor dysfunction in some patients with pheochromocytoma.

## Introduction

Adrenal pheochromocytomas are rare neuroendocrine tumors derived from chromaffin cells of the adrenal medulla [1]. These tumors synthesize and secrete catecholamines, including norepinephrine and epinephrine, into the bloodstream [1]. Excess catecholamine secretion can produce the classic triad of paroxysmal headaches, palpitations, and sweating, yet fewer than half of patients present with all 3 symptoms [2]. While hypertension was historically considered the hallmark feature of pheochromocytoma, the clinical presentation has shifted considerably in the modern era, with a significant proportion of patients now presenting with normal blood pressure [3]. The variable clinical presentation often delays diagnosis [3]. With the increasing use of routine cross-sectional imaging, approximately 61% of cases are now detected incidentally during imaging performed for unrelated indications [3].

Achalasia is a rare esophageal motility disorder resulting from progressive degeneration of inhibitory neurons in the myenteric plexus, leading to failure of the lower esophageal sphincter (LES) to relax [4]. Patients typically present with dysphagia, regurgitation of undigested food, nocturnal cough, chest pain, and weight loss [5]. While the precise etiology remains unknown, it is hypothesized to involve autonomic dysfunction and neurodegeneration within the enteric nervous system [4].

The overlap of symptoms between a pheochromocytoma and achalasia may delay recognition of the catecholamine-secreting tumor. Dysphagia, chest discomfort, and weight loss may be attributed to esophageal motility disorders, inadvertently masking adrenergic symptoms such as hypertension, palpitations, diaphoresis, and headaches. We hypothesize that adrenergic surges may contribute to the onset or progression of achalasia. We present a rare case in which evaluation for esophageal dysmotility in a patient with hypertension led to the discovery of an adrenal mass ultimately diagnosed as a pheochromocytoma.

## Case presentation

A 62-year-old female with a 7-year history of hypertension and a 2-year history of gastroesophageal reflux disease (GERD) presented with several months of progressive cough, dysphagia, and intermittent, nonbloody, nonbilious emesis. She also reported several episodes of night sweats and chills, and unintentional weight loss of 20 pounds over 2 years. She denied headaches, palpitations, chest pain, sleep disturbances, anxiety, or panic episodes.

Her home medications included hydrochlorothiazide-valsartan, famotidine, and omeprazole. She had no personal or family history of adrenal, renal, or malignant disease.

## Diagnostic assessment

On presentation, her blood pressure was 142/80 mmHg, and other vital signs were within normal limits. Pulmonary examination revealed crackles in the right lower lung field; the remainder of her physical examination was benign. Initial laboratory studies were unremarkable.

Chest radiography demonstrated multifocal alveolar opacities, right mid-to-upper lobe consolidation, and a dilated esophagus. A computerized tomography (CT) scan of the chest, abdomen, and pelvis revealed multifocal pneumonia, features suggestive of achalasia, and an incidental 5.7 cm left adrenal mass with an unenhanced CT attenuation of 115 Hounsfield units (HU) (Fig. 1A and 1B). The patient was admitted for further management of her pneumonia, gastrointestinal symptoms, and evaluation of the adrenal mass.

**Figure 1:**
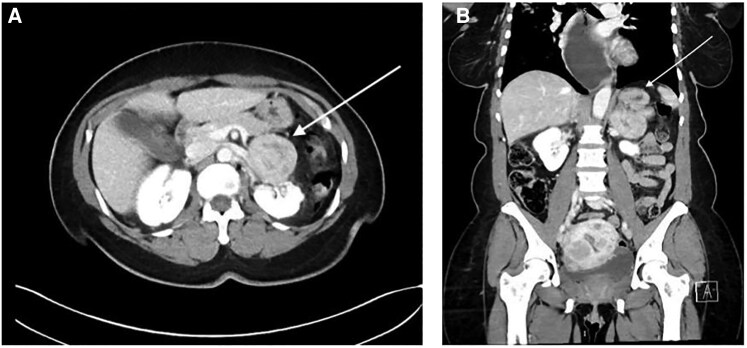
Contrast-enhanced computed tomography demonstrating a left adrenal mass (white arrow). (A) Axial view. (B) Coronal view.

Esophagogastroduodenoscopy showed marked esophageal dilation with retained food, consistent with achalasia. High-resolution manometry confirmed type II achalasia, and outpatient gastroenterology follow-up was planned for after the patient's discharge.

Abdominal magnetic resonance imaging (MRI) demonstrated a heterogeneous left adrenal mass with central necrosis. Biochemical evaluation of the adrenal mass included assessment for cortisol, aldosterone, and catecholamine excess. A 1-mg overnight dexamethasone suppression test demonstrated incomplete suppression, with a serum cortisol level of 2.3 µg/dL (SI: 63.4 nmol/L) (reference range, <1.8 µg/dL [SI: <50 nmol/L]). Plasma aldosterone concentration was 4 ng/dL (SI: 111 pmol/L) (reference range, 3-16 ng/dL [SI: 83-443 pmol/L]) with a plasma renin activity of 1.285 ng/mL/h (SI: 0.357 ng/mL/s) (reference range, 0.2-1.6 ng/mL/h [SI: 0.17-1.33 pmol/L/hr]) yielding an aldosterone-to-renin ratio of 3.1 (reference range, <20:1), excluding primary aldosteronism.

Plasma catecholamines and fractionated metanephrines were measured. Epinephrine was 20 pg/mL (SI 109.16 pmol/L) (reference range, <111 pg/mL [SI: <265 pmol/L]). Norepinephrine was 1875pg/mL (SI: 11 083 pmol/L) (reference range, 500-1700pg/mL [SI: 680–10 000 pmol/L]). Plasma normetanephrine was markedly elevated at 6400 pg/mL (SI: 35 100 pmol/L) (reference range, <148 pg/mL [SI: <1400 pmol/L]), while metanephrine was 30 pg/mL (SI: 152 pmol/L) (reference range <65 pg/mL [SI: <510 pmol/L]).

## Treatment

She completed a 7-day course of ceftriaxone and doxycycline for pneumonia. The patient subsequently underwent adrenalectomy following preoperative α-adrenergic blockade with doxazosin (2 mg orally once daily), initiated 17 days prior to surgery, and β-adrenergic blockade with metoprolol tartrate (12.5 mg orally twice daily), started 48 hours preoperatively.

Postoperatively, she was hemodynamically stable and in no distress.

Histopathological examination of the resected specimen demonstrated a 6.3 cm × 5.5 cm × 3.7 cm pheochromocytoma with small foci of capsular and vascular invasion. The tumor showed diffuse positivity for synaptophysin and chromogranin, with a Ki-67 proliferation index of approximately 1% to 2%. S100 immunostaining highlighted a well-preserved sustentacular cell network. All resection margins were free of tumor. Final pathologic staging was pT2, pN0, and pMx.

At discharge, her blood pressure was 134/87 mmHg, and physical examination was unremarkable. The patient was switched from nifedipine to amlodipine. Patient was scheduled for outpatient follow-up with endocrinology and gastroenterology services.

## Outcome and follow-up

At her 2-month follow-up, she reported complete resolution of night sweats and chills, with weight gain. Her blood pressure remained normal while continuing the same daily dose of amlodipine 10 mg. Repeat plasma catecholamines and metanephrines were within the reference range. The 1-mg overnight dexamethasone suppression test was repeated and was within the normal range. Comprehensive germline testing for hereditary syndromes associated with pheochromocytoma revealed no pathogenic variants in *MAX*, *RET*, *MEN1*, *SDHB*, *SDHD*, *CDC73*, *TMEM127*, *NF1*, *VHL*, *PTEN*, *SDHC*, *TP53*, or *PRKAR1A*.

She also demonstrated marked improvement in achalasia symptoms; however, she continued to experience mild dysphagia and nighttime cough, which she attributed to preexisting GERD. On repeat barium swallow fluoroscopy, there was improvement in the characteristic bird-beak morphology at the gastroesophageal junction, although residual narrowing of the lower esophageal sphincter persisted.

The patient subsequently underwent a Heller myotomy and fundoplication 4 months after adrenalectomy. At 6-month follow-up, she remained normotensive and asymptomatic, apart from a mild intermittent cough consistent with her chronic GERD.

## Discussion

Pheochromocytomas are rare tumors of adrenal or extra-adrenal chromaffin cells that secrete catecholamines [1]. Excess catecholamine secretion can produce the classic triad of paroxysmal headaches, palpitations, and sweating. Our patient, however, did not exhibit this triad. Her initial presentation was achalasia complicated by aspiration pneumonia, with pheochromocytoma incidentally identified on imaging and confirmed by a marked elevation in plasma normetanephrine (∼30-fold above the upper limit of normal), raising the possibility of an atypical association.

Achalasia typically presents with dysphagia, recurrent regurgitation of undigested food, respiratory symptoms such as nocturnal cough, chest pain, and weight loss [5]. Our patient initially presented with symptoms of achalasia, including persistent dysphagia, episodes of emesis, and chronic cough. She was also found to have unintentionally lost 20 pounds over several years. It is plausible that her gastrointestinal symptoms from achalasia masked the adrenergic manifestations of her pheochromocytoma, potentially delaying recognition of the underlying tumor.

Achalasia and pheochromocytomas can share overlapping symptoms. Both may cause chest pain, which in achalasia arises from esophageal spasm or distension [6], whereas in pheochromocytomas it results from cardiovascular changes induced by catecholamine excess, such as hypertension [1]. Both conditions can also present with unintentional weight loss. In achalasia, this typically occurs due to dysphagia or recurrent regurgitation [7], while in pheochromocytomas it is attributed to catecholamine-mediated effects on skeletal muscle metabolism [8]. Elevated normetanephrine levels have been shown to correlate more strongly with weight loss than other catecholamines [9]. Our patient exhibited markedly elevated normetanephrines consistent with this pattern.

Although an increasing number of cases have described pheochromocytoma presenting with gastrointestinal symptoms—including episodic nausea, vomiting, abdominal discomfort, and early satiety—it remains unclear whether these manifestations result from the local mass effect or atypical catecholamine-mediated mechanisms [9]. Majumder et al [10], in their discussion of nausea and its management in patients with pheochromocytoma, proposed stratifying tumors into 2 categories based on the risk of catecholamine crisis according to the biochemical profile. Our patient, with markedly elevated catecholamine secretion, would fall into the high-risk category for catecholamine crisis. This classification would limit the use of certain antiemetic therapies, such as dopamine agonists or corticosteroids, to avoid precipitating a catecholamine crisis.

Achalasia has been reported once as a paraneoplastic phenomenon in association with a neuroendocrine tumor; however, in that case, the proposed mechanism was autoimmune, mediated by antineuronal nuclear antibody-1, and definitive tumor resection was not performed, with management limited to botulinum toxin injections [11]. In contrast, in our case, the patient reported clinical improvement, and it was confirmed on repeat barium fluoroscopy, supporting our suggestion of a possible relationship between catecholamine excess from pheochromocytoma and esophageal motor dysfunction.

Achalasia is classically attributed to progressive loss of inhibitory nitrergic neurons within the myenteric plexus, resulting in impaired lower esophageal sphincter (LES) relaxation and increased sphincter tone [12]. Beyond enteric neurodegeneration, adrenergic mechanisms may also contribute. The cardioesophageal junction functions as a physiologic unit responsive to both sympathetic and vagal inputs [13]. Excess catecholamines stimulate α_1_-, α_2_-, and β_2_-adrenergic receptors on gastrointestinal smooth muscle while inhibiting cholinergic activity, leading to reduced peristalsis and increased sphincter contraction—mechanisms implicated in ileus and constipation and potentially relevant to achalasia [10, 14].

Chronic sympathetic activation may further alter receptor signaling through β-adrenergic receptor desensitization and downregulation [15]. Given that β_3_-adrenergic receptors mediate LES relaxation, their dysfunction could exacerbate impaired sphincter relaxation [16]. Additionally, norepinephrine, the precursor of normetanephrine—which was markedly elevated in our patient (∼30-fold above normal)—predominantly activates α-adrenergic receptors, whereas epinephrine has broader α- and β-adrenergic activity. Thus, the specific biochemical profile and magnitude of catecholamine excess in our patient may influence LES tone and contribute to the development or progression of achalasia.

In humans, spinal anesthesia or paravertebral sympathetic blockade has been shown to induce relaxation of the cardia, with complete resolution of achalasia (historically termed cardiospasm) reported in several cases [17]. Conversely, experimental α-adrenergic receptor stimulation via intravenous norepinephrine infusion increases LES pressure [16]. Taken together, these observations suggest that in the setting of markedly elevated circulating norepinephrine/normetanephrine, excessive adrenergic stimulation may contribute to reversible LES dysfunction. In this case, the copresentation of pheochromocytoma and achalasia, along with symptomatic improvement following adrenalectomy, suggests that excessive adrenergic stimulation may contribute to LES dysfunction or exacerbate preexisting motility abnormalities. However, because no other cases of this co-occurrence have been reported in the literature, the findings may also represent a coincidence. Further case reports and studies are needed to clarify whether a true association exists.

## Learning points

- Pheochromocytomas may present with atypical manifestations, including gastrointestinal dysmotility, and should be considered in patients with unexplained achalasia and an adrenal mass.
- Markedly elevated catecholamines, particularly norepinephrine, may contribute to reversible lower esophageal sphincter dysfunction via excessive adrenergic stimulation.
- Improvement in symptoms after adrenalectomy supports a potential association between catecholamine excess and esophageal motor dysfunction, highlighting the need for diagnostic vigilance and multidisciplinary evaluation.

## Contributors

All authors made individual contributions to authorship. G.R., J.W., and Z.Z. were involved in the diagnosis and management of this patient. S.I.M. provided supervision and contributed to the clinical interpretation and oversight of patient management. All authors reviewed and approved the final manuscript.

## Data Availability

Original data generated and analyzed during this study are included in this published article.
